# Diagnostic Testing Preferences in Rural and Vulnerable Populations During a Pandemic: Discrete Choice Experiment

**DOI:** 10.2196/68734

**Published:** 2025-10-21

**Authors:** Eline van den Broek-Altenburg, Jamie Benson, Yvonne Jonk, Abimbola Leslie, Jan Carney, Gary Stein

**Affiliations:** 1 Department of Radiology, College of Medicine, University of Vermont, 89 Beaumont Avenue, Burlington, VT, 05405, United States, 1 8024956029; 2 Perelman School of Medicine, University of Pennsylvania, Philadelphia, PA, United States; 3 Maine Rural Health Research Center, University of Southern Maine, Portland, OR, United States; 4 Department of Medicine, College of Medicine, University of Vermont, Burlington, VT, United States; 5 Department of Biochemistry, College of Medicine, University of Vermont, Burlington, VT, United States

**Keywords:** access to care, diagnostic testing, discrete choice experiment, individual and surveillance testing, pandemic, preferences, rural populations

## Abstract

**Background:**

A particular challenge during the COVID-19 pandemic was to provide testing and treatment for already disadvantaged and vulnerable populations. Many states implemented testing in a sporadic and disorganized way, and it is unclear to what extent this disproportionally affected population experienced barriers to accessing care. It is also unclear whether potential barriers to testing were caused by systemic challenges, such as rurality, or by individuals’ motivations for not getting tested.

**Objective:**

The objective of this study was to understand the trade-offs individuals in rural and vulnerable populations make between attributes of COVID-19 testing and how these vary across individuals. The study was part of RADx-UP, a consortium of more than 125 projects studying COVID-19 testing patterns in communities across the United States.

**Methods:**

First, we conducted 7 focus groups to identify barriers to COVID-19 testing and optimal strategies to increase testing. These barriers and strategies were then used to develop hypothetical choice scenarios in a discrete choice experiment. Data regarding preferences for testing were collected from an online panel (n=780) and oversampled in rural populations. We used quota sampling for age, gender, household income, and race: 50% of household incomes were above and below the median rural income of $52k per year 2023, and the maximum number of White, non-Hispanic respondents was 615. The data were analyzed using a conditional logit model (CL) and latent class analysis (LCA).

**Results:**

We found that the attributes for testing locations were almost all significant and had the expected signs. As hypothesized, respondents were less likely to choose a test location that had a higher wait time (coefficient −0.183, SE 0.006); more travel time to get tested (coefficient −1.129, SE0.054); that was higher cost (coefficient −0.020, SE 0.000); where someone else would collect the sample (coefficient −0.230, SE 0.036); where it would take more time to receive results (coefficient −0.032, SE 0.006); and where the tests would cause more discomfort (coefficient −0.125, SE 0.007). They were more likely to choose a mail-order option (coefficient 0.494, SE 0.075) and options that had higher test accuracy (coefficient 0.026, SE 0.001). While respondents cared about these structural factors, these were not the primary drivers of choice for testing. Some important covariates were driving preferences, including age, gender, medical vulnerability, insurance status, trust in government organizations, and previous flu vaccination, which may be a proxy for compliance. These covariates helped explain the observed preference heterogeneity.

**Conclusions:**

The results suggest that important social, behavioral, and policy factors affect choice for testing. Contrary to our hypotheses, rurality did not significantly impact preferences for testing; however, attitudes toward government and other beliefs did. Health care interventions intended to reduce rural health disparities that do not reflect the underlying values of individuals in those subpopulations are unlikely to be successful.

## Introduction

The COVID-19 pandemic created unprecedented challenges for health care and public health systems. Health care providers had to grapple with sudden changes in care delivery, including potential inpatient bed capacity constraints, delays in care, and the need to remotely manage medically and socially complex patients. Public health agencies had to quickly ramp up testing on an unparalleled scale; additionally, infrastructure and new processes to report these results to the public and health care providers were needed.

A particular challenge has been to provide COVID-19 testing and treatment for already disadvantaged and vulnerable populations [1-5], such as racial/ethnic disparities [6-11]. Media reports suggested socioeconomic and racial disparities in accessing COVID-19 testing. Many states implemented COVID-19 testing in a sporadic and disorganized way, partly because the Centers for Disease Control and Prevention guidelines changed several times over a short time span [12]. It is unclear to what extent disproportionately affected populations experienced barriers to accessing care. Even if historically underserved populations received fewer COVID-19 tests, the question remains whether this was due to systemic challenges or whether underlying individuals’ motivations influenced testing decisions.

Previous research has illustrated that many factors affect the formation of new habitual behavior, such as seeking a COVID-19 test. Recent data from a discrete choice experiment (DCE) suggested that the attribute of the highest relative importance was test result turnaround time, followed by the type of test, specimen, and venue [13]. A DCE provides the opportunity to estimate pairwise choices and analyze marginal values or the total value of a health service or good. In simulations by Zimba et al, immediate or same-day test results, both polymerase chain reaction and serology, or oral specimens substantially increased testing uptake over the current typical testing option. The simulated uptake of a hypothetical testing scenario of polymerase chain reaction and serology via a saliva sample at a pharmacy with same-day results was 97.7% in this study, compared to 1.8% opting not to test. This study was performed in 2020 when at-home tests were not on the market yet. Thus, the study did not take preference heterogeneity into account.

It remains unclear what other factors encourage behavior change and incentivize people to seek testing. A review of approaches across different fields reveals several shortcomings in public health policy, most importantly that public health interventions do not take into account various psychological and behavioral factors [13]. Five blocks of factors have been identified with regard to the new (health) behavior in the literature: risk, attitudinal, normative, ability, and self-regulation factors [14]. This aligns with the health belief model, which postulates that a person’s belief in a personal threat of an illness or disease, together with a person’s belief in the effectiveness of the recommended health behavior or action, such as COVID-19 testing, predicts the likelihood the person will adopt the behavior [15-17].

The aim of this study was to understand the trade-offs individuals in rural and vulnerable populations make between attributes of COVID-19 tests and how these vary across individuals. The study was conducted as part of RADx-UP, a consortium of more than 125 research projects studying COVID-19 testing patterns in communities across the United States and its territories, as well as Tribal Nations. The goal of RADx-UP is to “speed innovation in the development, commercialization, and implementation of technologies for COVID-19 testing.” The study was focused on identifying structural, social, behavioral, and policy factors that could be sources of COVID-19 testing disparities. Structural factors generally refer to broader conditions that either increase or decrease an individual’s likelihood of getting tested. In this study, the primary structural factor is access to testing, defined in terms of travel time, which has been found to be a strong predictor of satisfaction with access to care [18]. In addition to travel time, costs and waiting times can also be structural factors [13].

Our main hypothesis was that preferences for attributes of COVID-19 testing vary between and within subpopulations, particularly among vulnerable rural populations. We hypothesized that individuals were less likely to get tested if they faced longer travel wait times, more discomfort with testing, higher costs, or longer wait times to hear of test results. We also hypothesize that rurality is an important factor affecting preferences for testing, influencing the travel time and distance to the nearest testing center.

## Methods

### Overview

For this study, we used a mixed methods approach. To understand which aspects or attributes of testing were important to people, we first conducted a series of focus groups throughout the Northern New England region to identify barriers to COVID-19 testing among medically and socially vulnerable rural adult populations. We then identified optimal strategies to increase testing using hypothetical scenarios fielded within a representative sample of the rural US population by developing a DCE [19,20].

### Qualitative Research

We conducted 7 focus groups with 6-8 participants, each lasting approximately 45‐60 minutes. Interviews and focus groups were held in person or virtually via Zoom. Informed consent was obtained before all sessions, which were audio- or video-recorded and transcribed by a third-party professional transcription service. We used the 4-level classification scheme (Urban, Large Rural, Small Rural, and Isolated Small Rural-Categorization A) for the Rural-Urban Commuting Area codes, which is a census tract–based classification that uses standard census measures of population density, levels of urbanization, and journey-to-work commuting to characterize all US census tracts with respect to their rural/urban status and commuting relationships to other census tracts [21].

Collaboration with community organizations supported the recruitment of rural residents for the focus groups. Participants included older adults living in congregate housing and parents of school-aged children in rural communities. Focus group discussions explored participants’ trusted sources of health information, their decision-making processes for COVID-19 testing, and facilitators and barriers they encountered in accessing testing. These discussions provided insights into how rural communities navigated the information landscape during the COVID-19 public health crisis. Participants in the focus groups were offered a $30 gift card for their participation. Focus group transcripts were coded independently using NVivo v14.23.0 software and analyzed systematically using both deductive and inductive thematic analysis techniques.

The focus group questions addressed topics such as reasons for seeking COVID-19 testing, testing barriers, feelings about getting tested, history of vaccination hesitancy, and social distancing behaviors during the pandemic. We also asked about sources of information that influenced their decision-making process. Focus group discussions also explored region-specific testing logistics, common barriers, swapping methods available, trusted sources of health information, public health campaigns to increase testing, and resources facilitating access to testing.

### Experimental Design

The data from the focus groups identified distinct DCE terms and wording for conceptual attribute development [22]. Understanding how people made decisions about getting tested, and what sources of information informed their decisions, ensured the DCE accurately represented a realistic choice scenario. The unique contribution of a DCE is that it allows researchers to analyze the trade-offs that patients are willing to make, including options that may not exist but could in the future. DCEs are preferred to surveys because instead of simply asking “Would you get a COVID-19 test?”, this approach asks: “You have the choice between two tests, A and B; they differ in the following ways […]. Which would you prefer?” The set of direct, discrete choice options systematically varies and facilitates identifying and prioritizing the set of attributes that decision-makers care most about (eg, travel time vs swab testing method). DCEs follow a general pattern of describing a COVID-19 scenario, followed by questions to elicit underlying preferences. In this study’s DCE, testing is described by its attributes, and the options presented vary by the levels of those attributes. The DCE included 15 choice questions each, with 3 different testing options and an option not to test. The respondents were introduced to a hypothetical choice setting (see Multimedia Appendix 1) and asked to select which testing option they would prefer most.

Based on the qualitative research, we included 8 testing attributes: cost, travel time, wait time to results, wait time to test, test accuracy, testing venue, testing methods, and testing discomfort (see Multimedia Appendix 1 for details). We used a DCE in which we collected demographic data, data on housing and employment, vaccination acceptance, testing attitudes, risk-taking behaviors (using the GRiPS score, a validated general risk propensity scale [23]), conspiratorial thinking and anti-expert sentiment [24], trust in public health authorities [25], political preference, and religion. The marginal values of the testing attributes were estimated based on analyzing the set of pairwise choices [26]. Thus, the DCE facilitated analyzing trade-offs people were willing to make, including testing options that may not currently exist in the respondents’ local environments but could exist in the future.

**Figure 1.**
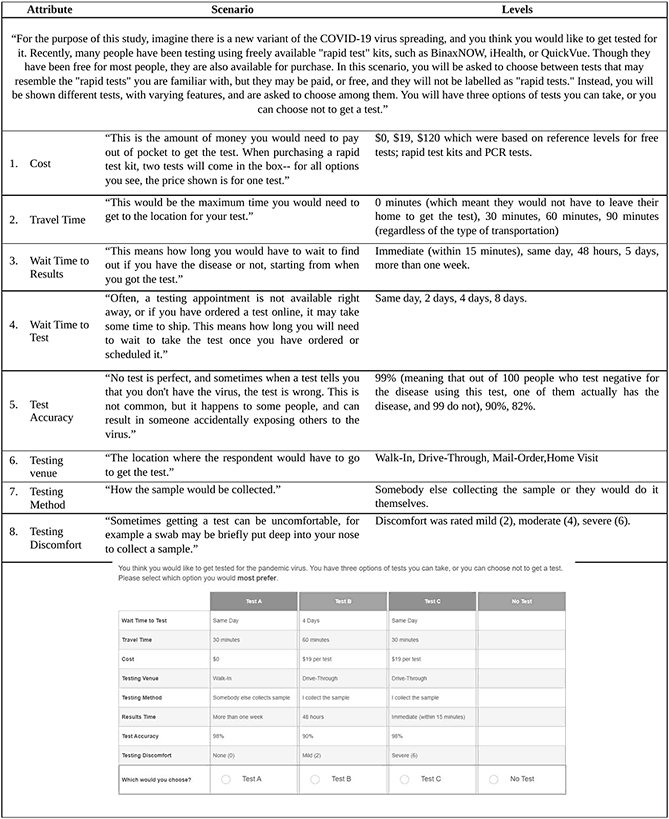
Choice attributes and an example of a choice task.

The experimental design of the DCE was based on a-priori estimates of the values respondents would give for different attributes of the choice. These numbers were based on pilot data and estimates from the literature. This approach allowed us to avoid using the full factorial design of all the options for attribute levels, but an “efficient” design that would optimize our information with just 15 combinations of attributes and levels. Figure 1 shows the attributes of a choice set and an example of a DCE choice set. The attributes and levels that differed by testing option were explained to participants prior to answering the survey questions with the vignette in Figure 1.

### Data

The 7 focus groups were conducted between November 2022 and March 2023. The DCE data were sampled from an online Centiment panel from April 29, 2023, through May 5, 2023. Centiment is a survey company that recruits individuals to answer surveys to generate rewards for themselves or to pledge their earnings to a nonprofit of their choice, and it is open to anyone to participate. Centiment has engineered complex systems to manage their respondents and ensure they are providing thoughtful responses. The standard invitation language for all members of the online Centiment panel is as follows: “We’ve got a survey for you. Reward: XX, Minutes: XX.” Centiment does not provide any detail beyond the associated reward and average time to complete to avoid any selection bias. Respondents who meet the targeting criteria will have the survey unlocked in their dashboard automatically and may come across it without an email. Initially, 1575 respondents initiated the survey but did not complete it. Out of 982 respondents who completed it, 184 were timed out before survey completion, leaving 798 completed surveys. Of the 798 respondents, 13 did not complete all the choice tasks, and 5 were likely robots based on our checks, leaving 780 completed responses. Of those 780 with good data, the mean survey duration was 28.25 minutes (median 20.52).

Centiment oversampled rural populations and used quota sampling for age, gender, household income, and race: 50% of household incomes were above and below the median rural income of $52k per year [27], and the maximum number of White, non-Hispanic respondents was 615. The study population included people who were medically or historically underserved: older adults in congregate housing, parents, and individuals living in Large Rural, Small Rural, and Isolated Small Rural areas nationally. The sample, therefore, included diverse and higher levels of social vulnerability and was nationally representative of rural populations. The online SurveyEngine platform was used to collect data (SurveyEngine GmbH, Berlin, Germany, 2023).

### Analytic Approach

We analyzed the data using both a CL model and LCA. The CL, developed by Daniel MacFadden [28], models the expected utilities in terms of characteristics of the alternatives rather than attributes of the individuals. The data from our repeated choice tasks can be treated as panel data in a conditional fixed effects logit model [29]. With this model, we used the respondents as their own controls and controlled for “stable” characteristics that do not change over time even if these were not measured. It is similar to an experiment with random assignment, controlling for omitted variables. With this model, we focused on the estimation of within-individual differences in preferences.

The latent class (LC) model is based on the assumption that individual preferences have a discrete distribution [30,31]. In the latter, we used a weighted probability of class membership and multiplied that with the probability of choosing a particular choice option. LC accounts for serial correlation, which means we model the probability of observing a sequence of choices. This way, we were able to predict the probability of the sequence of choices respondents made.

LC models are based on maximum likelihood estimation, adding information about the preference heterogeneity within our study population. For example, the LC model does not assume that everyone has the same preference for the no-choice option; rather, we analyzed whether some people were more likely to choose a no-choice option. Subgroup analysis can be used to identify heterogeneous treatment effects. LCA structure uses a class allocation model where probabilities vary across individual decision-makers as a function of their observed characteristics. Crucially, we analyzed whether membership in a subgroup may differ by health disparities, socioeconomic status, and different beliefs and attitudes by entering vulnerability, rural location, comorbidities, income, race, and other indicator variables in the class membership function.

We used the consistent Akaike information criterion (CAIC) and Bayesian information criterion (BIC), both statistical measures of fit, to determine the number of classes for the LCA [32]. Often the CAIC and BIC are used to determine the optimal number of classes, but with LC, we cannot directly compare the likelihood functions, as models with more parameters will generally produce better results, even though the model is not statistically considered better [33]. We used Stata 18.0 for the statistical analysis, which uses the expectation-maximization (EM) algorithm to fit a discrete-mixture logit model (StataCorp, College Station, TX, 2024). The EM algorithm is an iterative method to find (local) maximum likelihood or maximum a posteriori estimates of parameters in statistical models where the model depends on unobserved latent variables.

### Ethical Considerations

Focus group protocols were reviewed by the IRB using the exempt procedures set forth under 45 CFR 46.104, specifically, under Exemption Category: (2)(ii) tests, surveys, interviews, or observation (low risk), with waiver of documentation of consent under 46.117(c)(1). The Discrete Choice Experiment (DCE) was originally deemed not human subjects research by the IRB’s assessment tool; a quality review later determined that the study should have been reviewed and would likely have been approved with a waiver of written consent. In the DCE, survey data were collected without identifiers. Respondents received $5 compensation for their time from the survey company Centiment. Additionally, no identification of individual participants in any images of the manuscript or supplementary material is possible. The IRB director at UVM gave permission to publish this manuscript.

## Results

### Sample Characteristics

As described, data were gathered using a quota sampling approach for age, gender, household income, and race. We compared the sample characteristics of those who had previous COVID-19 tests to those who did not to see what the predicted uptake of testing would be, based on differences in attitudes and beliefs. Some studies have shown that previous choices may affect current choices, both in general terms [34] and in health choices specifically [35]. Within the descriptive analyses, chi-square tests were used to detect statistically significant differences between the two groups. Table 1 shows that those who had a previous COVID-19 test had a significantly higher GRiPS score and were somewhat more leaning toward politically liberal.

**Table 1.** Attitudes of respondents toward government and the public health system^a^.

Attitude	Previous COVID-19 test	
	No (n=206)	Yes (n=546)
Conspiratorial thinking score, mean (SD)	16.2 (6.3)	16.3 (6.3)
GRiPS risk score^b^, mean (SD)	17.1 (7.8)	17.7 (7.8)
Political leaning: conservative (1) to liberal (7)^b^, mean (SD)	3.2 (1.9)	3.7 (2.0)
Trust (0%‐100%), mean (SD)		
State’s government^b^	39.3 (32.4)	45.3 (30.9)
City/town government^b^	41.9 (32.6)	47.4 (30.4)
Police^b^	45.7 (34.0)	49.3 (33.7)
State health department^b^	49.0 (33.8)	56.8 (31.0)
Public health experts (CDC)^b^	48.3 (37.1)	55.8 (35.0)
Red Cross^b^	50.4 (34.6)	55.2 (33.1)
Health care system^b^	58.6 (32.8)	65.7 (30.7)
WHO^b^	41.4 (36.3)	48.7 (35.5)
Federal government^b^	31.5 (33.6)	37.9 (32.9)
Scientific researchers^b^	50.3 (35.1)	54.4 (34.4)
Governmental effort^b^	33.1 (31.9)	39.2 (32.1)
Religiosity, n (%)		
Atheistic/agnostic^b^	12 (5.8)	53 (9.7)
Organized religion^b^	102 (49.5)	282 (51.6)
Spiritual/non-organized/other^b^	92 (44.7)	211 (38.6)

a Data were collected from a discrete choice experiment eliciting preferences for COVID-19 testing from rural and vulnerable populations sampled from an online Centiment panel from April 29, 2023, through May 5, 2023.

b Significant difference between groups at P<.05.

Respondents who had a previous COVID-19 test also had more trust in public health organizations and governmental institutions, were more religious, and less spiritual than respondents who did not have a previous COVID-19 test. We did not find a statistical difference for conspiratorial thinking and anti-expert sentiment scores between people who had already gone for testing and those who did not.

### Conditional Logit

The results of the choice models can be found in Table 2. In the CL model, the dependent variable was “choice for test location,” defined by the attributes in the choice task. Each coefficient shows the probability of picking a particular option versus not picking that option. We found that the attributes were almost all significant and had the expected signs. As hypothesized, respondents were less likely to choose a test location that had a higher wait time (coefficient −0.183, SE 0.006); more travel time to get tested (−1.129, 0.054); that was more costly (−0.020, 0.000); where someone else would collect the sample (−0.230, 0.036); where it would take more time to receive results (−0.032, 0.006); and where tests would have more discomfort (−0.125, 0.007). They were more likely to choose a mail-order option (0.494, 0.075) and options that had higher test accuracy (0.026, 0.001).

**Table 2.** Results of the conditional logit model and latent class analysis—preferences for attributes of testing locations^a^.

	Conditional logit model β^b^ logit modelβ	Latent Class 1 “Compliant”	Latent Class 2 “Evaluative/less-compliant”	Latent Class 3 “Convenience”
Class share (%)	—^c^	0.434	0.159	0.407
Wait time to test (d)	−0.183^d^ (0.006)	−0.112^d^ (0.008)	−0.114^d^ (0.043)	−0.198^d^ (0.012)
Travel time to test (h)	−1.129^d^ (0.054)	−0.452^d^ (0.069)	−1.448^d^ (0.442)	−1.965^d^ (0.127)
Test cost ($)	−0.020^d^ (0.000)	−0.015^d^ (0.001)	−0.011^d^ (0.003)	−0.034^d^ (0.001)
Venue (ref: drive-through)				
Venue: walk-in	0.043 (0.040)	−0.079 (0.052)	0.205 (0.305)	−0.213^d^ (0.069)
Venue: home visit	0.061 (0.069)	−0.175(0.131)	0.373 (0.457)	−0.536^d^ (0.159)
Venue: mail order	0.494^d^( (0.075)	0.179^e^ (0.108)	0.871^f^( (0.443)	−0.567^d^ (0.134)
Test method	−0.230^d^ (0.036)	−0.275^d^ (0.047)	0.152 (0.254)	−0.130^f^ (0.061)
Time to results (d)	−0.032***^d^ (0.006)	−0.021***^d^ (0.008)	−0.030 (0.037)	−0.036^d^ (0.011)
Test accuracy (%)	0.026^d^ (0.001)	0.044^d^ (0.002)	0.024^d^ (0.005)	0.028^d^ (0.001)
Test discomfort	−0.125^d^ (0.007)	−0.030^d^ (0.009)	−0.131^d^ (0.046)	−0.163^d^ (0.021)

a Data were collected from a discrete choice experiment eliciting preferences for COVID-19 testing from rural and vulnerable populations sampled from an online Centiment panel from April 29, 2023, through May 5, 2023.

b The results show the coefficients (β) for the different attributes of the choice; standard errors in parenthesis.

c not applicable.

d *P*<.01.

e *P*<.10.

f *P*<.05.

### Latent Class Analysis

After identifying preferences for attributes of testing for the sample population, we addressed the issue of unobserved preferences of respondents by probabilistically segmenting the sample population into different groups or “classes” based on a latent variable. Class membership was first defined by a membership function including the indicator variables, after which the utility functions of different classes were estimated through maximum likelihood estimation. First, we determined the number of LC. We considered theoretical interpretability and compared the statistical tests of model fit using models for 1-5 possible LC.

There are several approaches to choosing the number of classes in LCA. When looking at the information criteria and the likelihood ratio test, there was a slightly better model fit for the 5-class model. Information criteria start with a computation of fit—the log-likelihood and then penalize this based on the number of classes. Information criteria commonly applied to the selection of number of classes include the BIC and the CAIC. However, other methods that are frequently used include replicability and domain-usefulness. The number of classes that get the most consistent results is considered to be the best. This approach can also be viewed as a form of cross-validation.

We combined this approach with domain usefulness, which means we choose the number of classes that create the solution that is most interesting from an interpretation perspective. This approach is often used when the difference in information criteria is small. The difference in model fit between the 3-, 4-, and 5-class models was not large, and the interpretability of the 3-class model is more straightforward. We therefore report the results of the 3-class model in Table 2.

The covariates we used for the class membership model included gender (female), education, income, age, race (White), currently insured, currently employed, self-assessed health rating, vulnerability, the number of previous tests, rurality, flu vaccination history, risk-taking score (GRiPS), conspiratorial thinking and anti-expert sentiment scales, trust in public health and government organizations, self-rated religiosity, and whether they were politically Republican-leaning.

Based on coefficients for the attributes of the choice for test location, three groups can be identified: testing method does not matter/drive-through locations are preferred, strong preference for at-home test and mail-order options, and prefers self-administering the test. We call the first class the “Compliant” class, since these respondents are more likely to get tested, but they do not care about the type of venue as long as they can take the test themselves. We call Class 2 the “Evaluative/less compliant” since they seem to care most about the ease of testing, not requiring time or travel, and do not care about the test method or how long it takes to get results back. The results implied that members of Class 2 would get tested because they had to or to be compliant, but not because they thought it was important. This suggests they only go for testing if they have to. Members of Class 3 want to drive somewhere for testing, but close to their homes. We call this class “Convenience.”

We found that age (−0.709, *P*<.01), being insured (1.742, *P*<.01), previous flu vaccination (0.580, *P*=.05), and trust in various institutions (0.510, *P*=.02) all significantly predicted membership of Class 1. For example, with every additional point increase on the Likert scale for trust, respondents would be more likely to be in Class 1 versus Class 3 which was the reference. We also found that gender (−2.610, *P*=.06), vulnerability (0.803, *P*=.030), previous flu vaccination (−0.921, *P*<.01), and trust in government institutions (−0.687, *P*=.04) also explained membership of Class 2. For example, females were far less likely than men to be in Class 2 compared to Class 3. Previous flu vaccination, which could be considered a proxy for “compliance,” defined Class 1 membership, but those who were vaccinated were less likely to be in Class 2. Where higher levels of trust in government institutions predicted Class 1 membership, these respondents were less likely to be in Class 2.

We found that 43.4% of the respondents in our study fall in Class 1, which is the group that cares significantly about being able to perform the test themselves. The coefficient (*β*=−.275) is negative and twice as large as for the other two groups, meaning that they would be less likely to pick a test location where someone else would collect the sample.

Class 2 includes 15.9% of respondents, and they seem to care the most about convenience, as they have a significant and positive coefficient for mail-order (*β*=.871). This seems to be more about not wanting to travel, since they do not feel strongly about who administers the test (*β*=.152). Indeed, the coefficient for travel time to the test center is large (*β*=−1.448) and negative, suggesting that with every additional hour of drive time, respondents are less likely to pick that testing option.

Class 3 has a strong preference for drive-through testing options. All the other venue options have a negative sign, meaning they are less likely to pick an option that involves walk-in (−0.213), home visit (−0.536), and mail-order (−0.567). The coefficient for travel time to test is large and negative (−1.965), meaning that with every 1-hour increase of travel time, respondents are disproportionately less likely to pick that testing option.

### Marginal Rates of Substitution

Table 3 shows an analysis of the trade-offs respondents were willing to make, known as the marginal rates of substitution. First, we looked at their willingness to pay for different attributes of testing. Members of all three classes were willing to pay significantly to decrease travel time. Class 1 members would pay $30 more to decrease travel time within 1 hour; this was $131 for members in Class 2 and $57 in Class 3. Members of the different classes were willing to pay for different venues but varied widely. For example, members of Class 2 were willing to pay $18 more for a walk-in testing option than drive-through, where they would be willing to pay $33 for an at-home testing option and $79 for mail order. Members of Class 1 would only pay more ($11) for a mail-order option, while members of Class 3 would not pay for any of these options when compared to drive-through. Members of Class 2 would also pay $11 more to receive a test with less discomfort, where this was only $2 for Class 1 and $4 for Class 3.

**Table 3.** Marginal rates of substitution when choosing for different diagnostic testing locations.

	WTP^a^ Class 1	WTP Class 2	WTP Class 3	Accuracy Class 1	Accuracy Class 2	Accuracy Class 3
Wait time to appointment	−7.445	−10.367	−5.822	2.530	−4.703	7.123
Travel time	−30.065	−131.631	−57.648	10.217	−59.715	70.537
Cost	—^b^	—	—	0.340	−0.454	1.224
Venue_walk	−5.251	18.655	−6.250	1.785	8.463	7.647
Venue_home	−11.639	33.933	−15.733	3.955	15.394	19.250
Venue_mail	11.913	79.194	−16.631	−4.049	35.927	20.350
Method	−18.320	13.845	−3.826	6.226	6.281	4.682
Time to results	1.428	−2.738	−1.048	−0.485	−1.242	1.282
Accuracy	2.943	−2.204	0.817	—	—	—
Discomfort	−2.014	−11.873	−4.788	0.685	−5.386	5.858

a WTP: willingness to pay.

b not applicable.

The table shows the marginal rates of substitution for costs (willingness to pay) and for the accuracy of the test for the 4 LC. For example, respondents in Class 1 were willing to pay $30 less if they had to travel 10 minutes more to a testing center.

Data were collected from a DCE eliciting preferences for COVID-19 testing from rural and vulnerable populations sampled from an online Centiment panel from April 29, 2023, through May 5, 2023.

In terms of test accuracy and results, only members of Classes 1 and 3 appeared to be willing to trade off wait time, travel time, and even discomfort to get a test that was more accurate. They were not willing to pay significantly more for a more accurate test, however. Members of Class 2 did not care about the accuracy of the test at all. This was consistent with findings for time to results: members of Class 2 would not trade off travel time, wait time, cost, or discomfort to get results more quickly. This also seems consistent with the covariates predicting class membership: these were respondents who had lower trust in government institutions, and fewer of them had previous flu vaccination.

## Discussion

### Principal Findings and Comparison With Previous Works

This study used data from a DCE to understand what trade-offs individuals in rural and vulnerable populations make between attributes of COVID-19, and how these vary by different individuals. We found that the attributes for testing locations were almost all significant and had the expected signs. As hypothesized, respondents were less likely to choose a test location that had a higher wait time (coefficient −0.183, SE 0.006); more travel time to get tested (−1.129, 0.054); that was more costly (−0.020, 0.000); where someone else would collect the sample (−0.230, 0.036); where it would take more time to receive results (−0.032, 0.006); and where tests would have more discomfort (−0.125, 0.007). They were more likely to choose a mail-order option (0.494, 0.075) and options that had higher test accuracy (0.026, 0.001). While we found that respondents cared about these structural factors, these were not the primary drivers of choice for testing. Some important covariates were driving preferences, including age, gender, medical vulnerability, insurance status, trust in government organizations, and previous flu vaccination—which may be a proxy for compliance. These covariates helped explain the observed preference heterogeneity.

It is essential to consider our findings regarding testing in the broader context of prior work on access to care, pandemic preparedness, strength of the public health workforce, and unique social and cultural factors potentially impacting health decisions. Several areas of public health practice and preparedness for future pandemics relate to our findings. Rurality in our study did not significantly impact testing preferences; however, the complexities and nuances in rural health differences are well documented [36-40]. Pandemic workforce stress resulted in an “exodus” of public health workers [38], leaving many areas of the United States vulnerable and unprepared for future pandemics. Additional literature further quantified workforce needs to stabilize state and local public health workforces to meet essential needs [39], including the necessity for “surge” capacity [40].

Published data from the Pew Research Center further demonstrate differences in urban, suburban, and rural residents’ political views on social issues, including health [41]. More recent studies highlight the variability of rural geographic settings, distinct rural classes, and the use of community capital to further define differences among rural communities [42].

A 2023 scoping review of COVID-19 preparedness in rural and remote areas showed many differences across broad social health determinants, including local economies and less health system preparation [43]. In contrast, local multisector collaborations, evidence of engaged local cultures, and the strength of local leaders were positive factors [43]. Similarly, the role of primary health care, adequacy of the public health local workforce, and strengths of local collaborations likely all play important roles in pandemic responses but may vary across rural geographic areas in the United States and be challenging to measure [44-46].

Other authors emphasize the importance of culture and history in rural communities, including cultural assets, social cohesion, “community spirit,” and the strengths of community leadership and engagement [47]. Strategies to optimize pandemic preparedness for the future (in addition to adequate health care and public health workforce) must incorporate familiarity with, engagement with, and respect for these factors to fully leverage optimal responses to public health threats, including, but not limited to, COVID-19. Given the complexity of local community culture, it is not surprising that it is challenging to systematically increase access to testing: our study helps identify actionable priorities to help guide future public health interventions.

While the findings of this study add significantly to the existing literature regarding access to diagnostic testing during the pandemic, they are not without limitations. The study was limited by its relatively small sample size and snowball sampling technique. The COVID-19 pandemic is an evolving crisis, and this research was only focused on a particular period of time which we studied at a later period of time. Nevertheless, many study participants said they clearly remembered their considerations at the time, since it was relatively recent. The hypothetical setting of the DCE and the online panel makes it challenging to make out-of-sample predictions, which is why our conclusions cannot be generalized to potential future pandemic scenarios.

It is important to note from the results of the LCA that decision-makers looking to optimize testing strategies, while incorporating patient preferences for attributes of testing, should differentiate between the different subpopulations they are serving. This requires careful consideration of individual characteristics as well as preferences. Overall, we found in this study that subpopulations are characterized by being either primarily compliant or evaluative to some extent non-compliant, or they were driven solely by convenience. We did not find an association between rurality and testing preferences, which means that rurality was not associated with the probability of belonging to a specific class. It does not mean such an association does not exist. An alternative to investigate such interaction is with a mixed logit model, which allows for individual intercepts and more flexible substitution patterns. However, the sample size was not significantly large to analyze elaborate interaction models. This would be important to further analyze in future studies.

### Conclusions

This suggests that important social, behavioral, and even policy factors affect choice for testing. Contrary to our hypotheses, rurality did not significantly impact preferences for testing. This study provides a clear message to public health and surveillance systems seeking to increase testing rates during a pandemic like COVID-19. We conclude that health care interventions intended to reduce rural health disparities that do not reflect the underlying values of individuals in those subpopulations are unlikely to be successful.

Another concrete public health implication from this study is that adding a consistent and “easy” mail-order testing option in a future pandemic may significantly increase testing rates. Both the total sample population in the CL model and the different classes identified in the LCA, while having different underlying tastes and unobserved preferences, have a significant, positive, and relatively large effect size for adding this hypothetical mail-order option. Members of two or three classes, adding up to 84% of all respondents, also had a strong preference for self-administering the test. Compared to other attributes of testing, such as travel time or wait time, mail-order and self-administering are significantly more important aspects of testing for most people.

## Supplementary material

10.2196/68734 Multimedia Appendix 1 Hypothetical choice setting and choice attributes.

## Abbreviations

BIC: Bayesian information criterion
CAIC: consistent Akaike information criterion
CL: conditional logit
DCE: discrete choice experiment
EM: expectation-maximization
LC: latent class
LCA: latent class analysis
