# Correction: Diagnostic Testing Preferences in Rural and Vulnerable Populations During a Pandemic: Discrete Choice Experiment

**DOI:** 10.2196/87714

**Published:** 2026-03-13

**Authors:** Eline van den Broek-Altenburg, Jamie Benson, Yvonne Jonk, Abimbola Leslie, Jan Carney, Gary Stein

**Affiliations:** 1 Department of Radiology, College of Medicine University of Vermont Burlington, VT United States; 2 Perelman School of Medicine University of Pennsylvania Philadelphia, PA United States; 3 Maine Rural Health Research Center University of Southern Maine Portland, OR United States; 4 Department of Medicine College of Medicine University of Vermont Burlington, VT United States; 5 Department of Biochemistry College of Medicine University of Vermont Burlington, VT United States

In “Diagnostic Testing Preferences in Rural and Vulnerable Populations During a Pandemic: Discrete Choice Experiment” [1], the authors noted one error.

The Ethical Considerations section has been changed from the following:

This study (STUDY00002116) has been reviewed by the Institutional Review Board of the University of Vermont and the Institutional Official has authorized the publication of the research data.. Focus group protocols were reviewed by the IRB using the exempt procedures set forth under 45 CFR 46.104, specifically, under Exemption Category: (2)(ii) tests, surveys, interviews, or observation (low risk), with waiver of documentation of consent under 46.117(c)(1). The Discrete Choice Experiment (DCE) was originally deemed not human subjects research by the IRB’s assessment tool; a quality review later determined that the study should have been reviewed and would likely have been approved with a waiver of written consent. In the DCE, survey data were collected without identifiers. Respondents received $5 compensation for their time from the survey company Centiment. Additionally, no identification of individual participants in any images of the manuscript or supplementary material is possible.

This section now reads:

Focus group protocols were reviewed by the IRB using the exempt procedures set forth under 45 CFR 46.104, specifically, under Exemption Category: (2)(ii) tests, surveys, interviews, or observation (low risk), with waiver of documentation of consent under 46.117(c)(1). The Discrete Choice Experiment (DCE) was originally deemed not human subjects research by the IRB’s assessment tool; a quality review later determined that the study should have been reviewed and would likely have been approved with a waiver of written consent. In the DCE, survey data were collected without identifiers. Respondents received $5 compensation for their time from the survey company Centiment. Additionally, no identification of individual participants in any images of the manuscript or supplementary material is possible. The IRB director at UVM gave permission to publish this manuscript.

The correction will appear in the online version of the paper on the JMIR Publications website, together with the publication of this correction notice. Because this was made after submission to PubMed, PubMed Central, and other full-text repositories, the corrected article has also been resubmitted to those repositories.
