# Supplementary material for: Diagnostic Testing Preferences in Rural and Vulnerable Populations During a Pandemic: Discrete Choice Experiment
Source: JMIR Public Health Surveill. 2025 Oct 21;11:e68734. doi: 10.2196/68734 (PMC12539797; doi:10.2196/68734)
Supplement: Multimedia Appendix 1 [file publichealth-v11-e68734-s001.docx]

**Appendix**

Based on the qualitative research, we included 8 attributes of the choice: cost, travel time, wait time to results, test accuracy, testing venue, testing methods and testing discomfort.

- Cost - which was described as “this is the amount of money you would need to pay out of pocket to get the test. When purchasing a rapid test kit, two tests will come in the box-- for all options you see, the price shown is for one test.” The possible levels of the cost attribute were: $0, $19, $120 which were based on reference levels for free tests; rapid test kits and PCR tests.
- Travel Time – “this would be the maximum time you would need to get to the location for your test”. The levels included 0 minutes -which meant they would not have to leave their home to get the test-, 30 minutes, 60 minutes, 90 minutes. test (regardless of the type of transportation)
- Wait Time to Results – “This means how long you would have to wait to find out if you have the disease or not, starting from when you got the test”. The options included Immediate (within 15 minutes), same Day, 48 hours, 5 days, more than one week.
- Wait Time to Test – which was describes as “often, a testing appointment is not available right away, or if you have ordered a test online, it may take some time to ship. This means how long you will need to wait to take the test once you have ordered or scheduled it”. The levels were same day, 2 days, 4 days, 8 days.
- Test Accuracy which was described as “No test is perfect, and sometimes when a test tells you that you don't have the virus, the test is wrong. This is not common, but it happens to some people, and can result in someone accidentally exposing others to the virus”. The options included 99% (meaning that out of 100 people who test negative for the disease using this test, one of them actually has the disease, and 99 do not), 90%, 82%.
- Testing venue which meant the location where respondent would have to go to get the test, which included the option “Walk-In”, “Drive-Through”, “Mail-Order”, “Home Visit”.
- ﻿Testing Method which meant how the sample would be collected which could be either somebody else collecting the sample or they would do it themselves.
- Testing Discomfort which was described as “sometimes getting a test can be uncomfortable, for example a swab may be briefly put deep into your nose to collect a sample. Discomfort was rated on from mild (2), moderate (4), severe (6).

The respondents were introduced to the hypothetical choice setting in the following way:

*“For the purpose of this study, imagine there is a new variant of the COVID-19 virus spreading, and you think you would like to get tested for it. Recently, many people have been testing using freely available "rapid test" kits, such as BinaxNOW, iHealth, or QuickVue. Though they have been free for most people, they are also available for purchase. In this scenario, you will be asked to choose between tests that may resemble the "rapid tests" you are familiar with, but they may be paid, or free, and they will not be labelled as "rapid tests." Instead, you will be shown different tests, with varying features, and are asked to choose among them. You will have three options of tests you can take, or you can choose not to get a test.”*
